# Supplementary material for: Functional Outcomes in Head and Neck Cancer Patients
Source: Cancers (Basel). 2022 Apr 25;14(9):2135. doi: 10.3390/cancers14092135 (PMC9099625; doi:10.3390/cancers14092135)
Supplement: Supplementary file 1 [file cancers-14-02135-s001.zip › cancers-1661528-supplementary.pdf]

cancers

MDPI

Supplementary files

Functional outcomes in head and neck cancer patients

Functional domain	Integrity Grade					Due to tumor / treatment
	0	1	2	3	4	
**Food intake**	No oral feeding; only via gastrostomy tube	Gastrostomy tube needed; some oral feeding possible	No gastrostomy tube, oral diet, but only liquid/soft food	No gastrostomy tube, diet/ swallowing near normal	normal	
**Breathing**	Tracheostoma, needs blocked cannula	Tracheostoma, speech cannula/no cannula	No tracheostoma, breathing difficulties at rest	No tracheostoma, breathing difficulties only on exertion	normal	
**Speech**	Not possible, without phonation	Difficult to understand, no phone calls	Difficult to understand, phone calls possible	Easy to understand, but pronunciation/ voice changed	normal	
**Pain**	Pain despite opiate therapy	Controlled with opiates	Regularly needs non-opioid analgesics	Needs analgesics from time to time	normal	
**Mood**	Suicidal thoughts	Very depressed despite antidepressants	with antidepressants overall normal mood, very depressed without antidepressants	Occasionally depressed, no antidepressants needed	normal	
**Neck & shoulder mobility**	Stiff neck and/or shoulder, hardly any movement possible	Can hardly comb hair, looking backwards in car not possible	Combing with problems, looking backwards in car difficult	Combing and looking backwards in car slightly restricted	normal	

Figure S1. The Head and Neck Carcinoma Functional InTegrity (HNC-FIT) Scales are a matrix of 6 verbal rating scales with 5 levels reflecting the functional level of food intake, breathing, speech, pain, mood and neck and shoulder mobility. In this matrix, the respective functional levels are marked when the investigator takes the interim oncologic history.

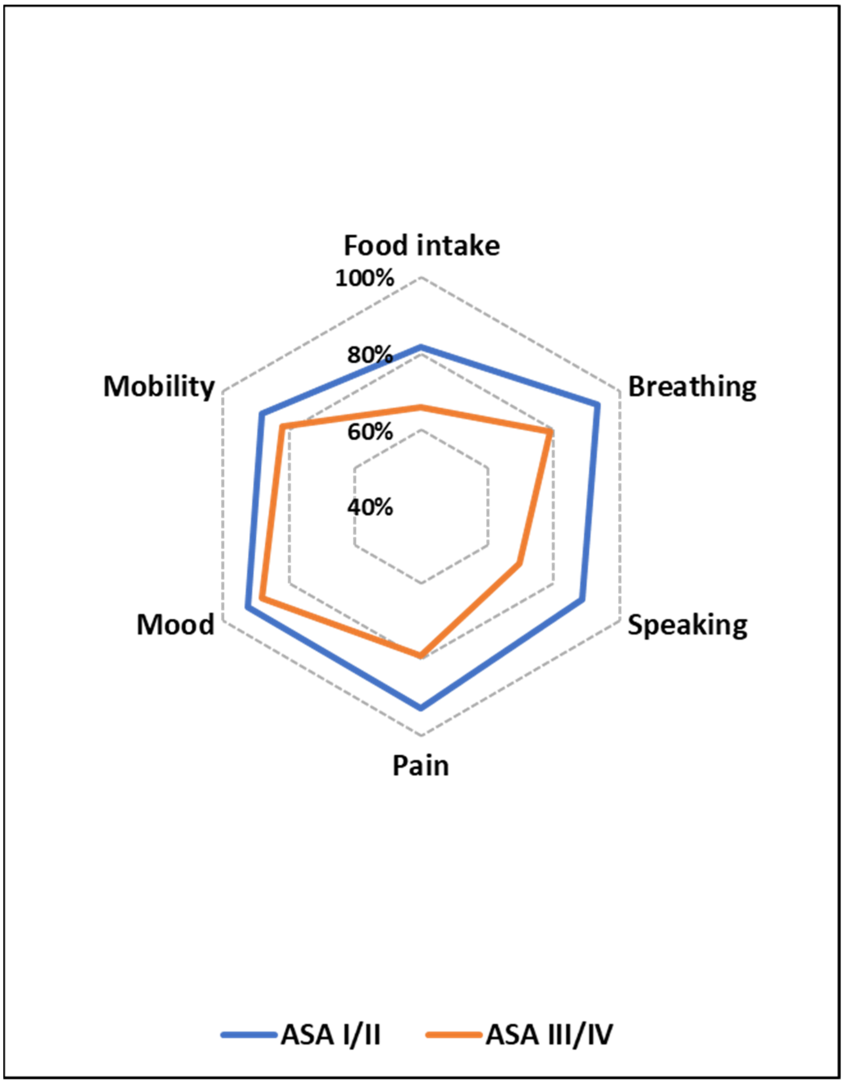

Figure S2. Star plot representing percentage (star axes) of HNC-patients with normal or near-normal functional outcome (functional integrity) in 6 functional domains depending on general health status. American Society of Anesthesiology (ASA) physical status score served as indicator for general health status and dichotomized into ASA I/II vs. ASA III/IV.

Suppl. text S1. Instructions for clinicians on how to complete the Head and Neck Cancer Functional InTegrity Scales.

The functional domain food intake includes eating and drinking. ‘Unable to swallow; only via gastrostomy tube’ means that the patient is unable to swallow, and his intake of food is exclusively via gastrostomy (or nasogastral) tube. This applies also for nasogastric tubes. ‘Gastrostomy tube needed, some oral feeding possible’ means that the patient relies on PEG for adequate food and fluid intake, but occasional sips are possible. ‘No gastrostomy tube, oral diet, but only liquid/soft food’ means that the patient does not have a gastrostomy tube but cannot eat solid food. ‘No gastrostomy tube, diet near normal’ means that the patient can essentially eat normally, but with certain (e.g. dry foods) problems, but these can be overcome (for example, by simultaneously taking liquid). ‘Normal’, like in all functional domains, means that the function is as it was before the illness.

In the functional domain respiration 'Tracheostoma, needs blocked cannula' means that the patient has a tracheostoma and must use a cannula with blocked cuff, e.g. because of aspiration. ‘Tracheostoma, speech cannula/no cannula’ means a simple condition with tracheostomy. ‘No tracheostoma, breathing difficult at rest’ is ticked at dyspnea at rest. With ‘No tracheostoma, breathing difficulties only on exertion’, a typical loading situation would be e.g. climb stairs.

In the functional domain speech ‘Not possible, without phonation’ means that the patient is incapable of speech and essentially must rely on written communication. At 'Difficult to understand, no phone calls', the patient can indeed make sounds and you can understand the meaning with high concentration, but the patient in not able to make calls. At 'Telephoning possible' is the language is very limited and difficult to understand, but it is a communication over the phone with strangers possible. ‘Easy to understand, but pronunciation/voice changed’ also detects slight functional limitations of speech, but the communication is possible without any problems.

In the pain dimension ‘pain despite of opiate therapy" means that the patient suffers from pain despite pain therapy with opiates by experienced pain specialists. 'Controlled with opiates' means that with adequate pain therapy including opiates the patient is essentially painless. 'Regularly needs non-opioid analgesics' means that the patient has a long-term pain therapy without opioids and is thus essentially painless. 'Needs analgesics from time to time' means occasional pain and occasionally use of painkillers.

In the dimension mood, 'suicidal thoughts' means that the patient is so depressed that he has suicidal thoughts. As a rule, psychiatric intervention is urgently needed. 'Very depressed despite antidepressants' means that the patient has sustained depressive mood despite adequate antidepressant therapy received by a suitable medical facility. 'With antidepressants overall normal mood' means that the patient regularly needs antidepressants because of depressive mood. With this antidepressant therapy, however, the mood is essentially normal. 'Occasionally depressed, no antidepressants needed' is ticked in case of occasional depressive mood.

In the shoulder-neck-mobility dimension two functions are queried simultaneously, namely shoulder mobility and neck mobility. In each case the worse functional status is ticked. ‘Stiff neck and/or shoulder, hardly any movement possible" is a complete fixation in the neck and/or shoulder area. 'Can hair hardly comb, looking backwards in car not possible' means that the shoulder mobility is so severely limited that with a straight head not all areas of the head are reachable with the comb and/or that without a rear view camera the car cannot be reversed under visual control because the head cannot be turned far enough. 'Combing with problems, looking backwards in car difficult' means that

combing the hair when the head is straight and that reversing under sight is possible, but means a considerable effort. 'Combing and looking backwards in car near normal' means a slight restriction of the neck and shoulder mobility without significant functional impairment.

Table S1. Results of binary logistic regression including response status at last follow up for the functional domain **food intake**. Odds ratios (Exp(B)) with 95% confidence intervals are provided. Reference groups were: Gender female, age group >80 years; ASA III/IV; smoking <10 PY; alcohol consumption <daily; tumor stage T4, tumor site hypopharynx; treatment modality primary ST/RT; p16 negative.

		B	S.E.	Wald	df	Sig.	Exp(B)	95%CI for Exp(B)	
								Lower	Upper
**Step 1[a]**	Age groups at first diagnosis			5.328	4	0.255			
	Age groups at first diagnosis (1)	0.378	0.952	0.158	1	0.691	1.460	0.226	9.439
	Age groups at first diagnosis (2)	-0.078	0.869	0.008	1	0.928	0.925	0.168	5.079
	Age groups at first diagnosis (3)	-0.555	0.867	0.410	1	0.522	0.574	0.105	3.141
	Age groups at first diagnosis (4)	-0.552	0.875	0.398	1	0.528	0.576	0.103	3.201
	ASA (1)	0.809	0.288	7.872	1	0.005	2.246	1.276	3.953
	Smoker (1)	0.634	0.284	4.961	1	0.026	1.884	1.079	3.291
	Drinking (1)	0.412	0.291	1.999	1	0.157	1.509	0.853	2.670
	T stage truncated			11.434	3	0.010			
	T stage truncated (1)	1.480	0.565	6.865	1	0.009	4.391	1.452	13.279
	T stage truncated (2)	1.544	0.475	10.564	1	0.001	4.683	1.846	11.883
	T stage truncated (3)	1.038	0.471	4.856	1	0.028	2.825	1.122	7.115
	N stage truncated			5.133	3	0.162			
	N stage truncated (1)	1.117	0.564	3.928	1	0.047	3.056	1.012	9.227
	N stage truncated (2)	1.019	0.579	3.096	1	0.078	2.770	0.890	8.618
	N stage truncated (3)	0.547	0.718	0.580	1	0.446	1.728	0.423	7.053
	UICC truncated			4.884	3	0.181			
	UICC truncated (1)	-0.045	0.905	0.002	1	0.960	0.956	0.162	5.637
	UICC truncated (2)	-1.332	0.705	3.566	1	0.059	0.265	0.066	1.052
	UICC truncated (3)	-0.604	0.644	0.880	1	0.348	0.547	0.155	1.931
	Common tumor sites			12.699	4	0.013			
	Common tumor sites (1)	-0.849	0.561	2.291	1	0.130	0.428	0.143	1.284
	Common tumor sites (2)	-0.146	0.464	0.099	1	0.754	0.864	0.348	2.147
	Common tumor sites (3)	0.713	0.541	1.735	1	0.188	2.040	0.706	5.893
	Common tumor sites (4)	0.724	0.686	1.113	1	0.291	2.063	0.537	7.922
	Treatment modalities			10.055	4	0.040			
	Treatment modalities (1)	1.166	0.456	6.528	1	0.011	3.210	1.312	7.853
	Treatment modalities (2)	0.219	0.350	0.392	1	0.531	1.245	0.627	2.470
	Treatment modalities (3)	-0.537	0.447	1.440	1	0.230	0.585	0.243	1.405
	Treatment modalities (4)	0.342	0.684	0.251	1	0.617	1.408	0.368	5.383
	Constant	-1.275	1.050	1.473	1	0.225	0.280		
**Step 4[a]**	ASA (1)	0.823	0.269	9.333	1	0.002	2.276	1.343	3.858
	Smoker (1)	0.484	0.271	3.191	1	0.074	1.622	0.954	2.759
	Drinking (1)	0.511	0.281	3.300	1	0.069	1.667	0.960	2.895
	T stage truncated			14.372	3	0.002			
	T stage truncated (1)	1.434	0.419	11.683	1	0.001	4.194	1.843	9.543
	T stage truncated (2)	1.062	0.338	9.882	1	0.002	2.892	1.492	5.609
	T stage truncated (3)	0.684	0.375	3.338	1	0.068	1.982	0.951	4.131
	Common tumor site			15.610	4	0.004			
	Common tumor site (1)	-0.518	0.530	0.955	1	0.328	0.596	0.211	1.684
	Common tumor site (2)	0.055	0.446	0.015	1	0.902	1.057	0.441	2.531
	Common tumor site (3)	1.049	0.517	4.117	1	0.042	2.855	1.036	7.867
	Common tumor site (4)	1.158	0.664	3.036	1	0.081	3.183	0.865	11.704
	Treatment modalities			13.943	4	0.007			

Treatment modalities (1)	1.308	0.399	10.755	1	0.001	3.699	1.693	8.085
Treatment modalities (2)	0.324	0.328	0.977	1	0.323	1.383	0.727	2.633
Treatment modalities (3)	-0.404	0.433	0.874	1	0.350	0.667	0.286	1.558
Treatment modalities (4)	0.339	0.620	0.299	1	0.584	1.404	0.416	4.733
Constant	-1.360	0.521	6.817	1	0.009	0.257		

[a] Variables(s) entered on step 1: Age groups at first diagnosis, ASA, Smoker, Drinking, T stage truncated, N stage truncated, UICC truncated, Common tumor sites, Treatment modalities.

Table S2. Results of binary logistic regression including response status at last follow up for the functional domain **breathing**. Odds ratios (Exp(B)) with 95% confidence intervals are provided. Reference groups were: Gender female, age group >80 years; ASA III/IV; smoking <10 PY; alcohol consumption <daily; tumor stage T4, tumor site hypopharynx; treatment modality primary ST/RT; p16 negative.

		B	S.E.	Wald	df	Sig.	Exp(B)	95%CI for Exp(B)	
								Lower	Upper
**Step 1**[a]	Gender (1)	**0.301**	0.588	0.003	1	0.959	1.031	0.326	3.266
	ASA (1)	1.026	0.417	6.070	1	0.014	2.790	1.234	6.313
	Smoker (1)	0.428	0.435	0.967	1	0.325	1.534	0.654	3.596
	Common tumor sites			35.783	4	0.000			
	Common tumor sites (1)	3.379	0.892	14.348	1	0.000	29.353	5.108	168.681
	Common tumor sites (2)	3.239	0.613	27.874	1	0.000	25.506	7.664	84.885
	Common tumor sites (3)	1.245	0.537	5.365	1	0.021	3.471	1.211	9.951
	Common tumor sites (4)	3.189	1.159	7.576	1	0.006	24.266	2.505	235.074
	T stage truncated			11.115	3	0.011			
	T stage truncated (1)	2.297	1.167	3.873	1	0.049	9.945	1.009	97.973
	T stage truncated (2)	1.870	0.705	7.028	1	0.008	6.486	1.628	25.840
	T stage truncated (3)	0.078	0.585	0.018	1	0.895	1.081	0.343	3.401
	UICC truncated			0.146	3	0.986			
	UICC truncated (1)	0.082	1.317	0.004	1	0.950	1.086	0.082	14.346
	UICC truncated (2)	-0.295	0.826	0.127	1	0.721	0.745	0.148	3.761
	UICC truncated (3)	0.003	0.606	0.000	1	0.996	1.003	0.306	3.291
	p16_Kat(2) (1)	0.861	0.623	1.907	1	0.167	2.365	0.697	8.020
	Treatment modalities			3.741	4	0.442			
	Treatment modalities (1)	-0.902	0.632	2.036	1	0.154	0.406	0.117	1.401
	Treatment modalities (2)	-0.797	0.547	2.121	1	0.145	0.451	0.154	1.317
	Treatment modalities (3)	-0.949	0.785	1.461	1	0.227	0.387	0.083	1.803
	Treatment modalities (4)	-1.208	0.796	2.304	1	0.129	0.299	0.063	1.421
	Constant	-1.145	0.798	2.059	1	0.151	0.318		
**Step 5**[a]	ASA (1)	1.080	0.392	7.580	1	0.006	2.945	1.365	6.354
	Common tumor site			38.617	4	0.000			
	Common tumor site (1)	3.254	0.844	14.853	1	0.000	25.886	4.948	135.418
	Common tumor site (2)	3.215	0.595	29.187	1	0.000	24.913	7.759	79.990
	Common tumor site (3)	1.119	0.497	5.064	1	0.024	3.061	1.155	8.112
	Common tumor site (4)	3.078	1.123	7.521	1	0.006	21.723	2.407	196.065
	T stage truncated			17.641	3	0.001			
	T stage truncated (1)	2.001	0.615	10.586	1	0.001	7.395	2.216	24.684
	T stage truncated (2)	1.372	0.529	6.714	1	0.010	3.942	1.397	11.123
	T stage truncated (3)	0.026	0.495	0.003	1	0.958	1.027	0.389	2.707
	p16_Kat(2) (1)	1.018	0.604	2.843	1	0.092	2.768	0.848	9.038
	Constant	-1.413	0.545	6.731	1	0.009	0.243		

[a] Variables(s) entered on step 1: Gender, ASA, Smoker, Common tumor site, T stage truncated, UICC truncated, p16_Kat2, Treatment.

Table S3. Results of binary logistic regression including response status at last follow up for the functional domain **speech**. Odds ratios (Exp(B)) with 95% confidence intervals are provided. Reference groups were: Gender female, age group >80 years; ASA III/IV; smoking <10 PY; alcohol consumption <daily; tumor stage T4, tumor site hypopharynx; treatment modality primary ST/RT; p16 negative.

		B	S.E.	Wald	df	Sig.	Exp(B)	95%CI for Exp(B)	
								Lower	Upper
	Gender (1)	**-0.764**	0.519	2.169	1	0.141	0.466	0.169	1.287
**Step 1**[a]	Age groups at first diagnosis			0.763	4	0.943			
	Age groups at first diagnosis (1)	0.535	0.911	0.345	1	0.557	1.708	0.287	10.180
	Age groups at first diagnosis (2)	0.114	0.772	0.022	1	0.882	1.121	0.247	5.087
	Age groups at first diagnosis (3)	0.079	0.777	0.010	1	0.919	1.082	0.236	4.960
	Age groups at first diagnosis (4)	0.285	0.773	0.136	1	0.713	1.329	0.292	6.046
	ASA (1)	0.965	0.338	8.148	1	0.004	2.621	1.352	5.080
	Smoker (1)	0.000	0.341	0.000	1	0.999	1.000	0.513	1.952
	Common tumor sites			21.007	4	0.000			
	Common tumor sites (1)	1.890	0.666	8.052	1	0.005	6.619	1.794	24.416
	Common tumor sites (2)	1.719	0.521	10.865	1	0.001	5.578	2.007	15.499
	Common tumor sites (3)	0.471	0.522	0.817	1	0.366	1.602	0.576	4.455
	Common tumor sites (4)	3.182	1.167	7.432	1	0.006	24.084	2.445	237.214
	T stage truncated			19.976	3	0.000			
	T stage truncated (1)	3.285	1.100	8.757	1	0.003	26.706	3.032	235.239
	T stage truncated (2)	2.273	0.601	14.324	1	0.000	9.706	2.992	31.493
	T stage truncated (3)	0.649	0.496	1.716	1	0.190	1.914	0.725	5.058
	UICC truncated			1.096	3	0.778			
	UICC truncated (1)	-0.860	1.173	0.538	1	0.463	0.423	0.042	4.211
	UICC truncated (2)	-0.284	0.673	0.178	1	0.673	0.753	0.202	2.813
	UICC truncated (3)	0.222	0.529	0.176	1	0.675	1.248	0.443	3.518
	p16_Kat2 (1)	0.616	0.451	1.870	1	0.171	1.852	0.766	4.480
	Treatment modalities			13.857	4	0.008			
	Treatment modalities (1)	-1.612	0.566	8.112	1	0.004	0.199	0.066	0.605
	Treatment modalities (2)	-0.645	0.483	1.783	1	0.182	0.525	0.203	1.352
	Treatment modalities (3)	-1.889	0.628	9.138	1	0.003	0.150	0.044	0.513
	Treatment modalities (4)	-0.403	0.737	0.300	1	0.584	0.668	0.158	2.830
	Constant	-0.418	1.004	0.174	1	0.677	0.658		
**Step 5**[a]	Gender (1)	-0.819	0.508	2.596	1	0.107	0.441	0.163	1.194
	ASA (1)	1.024	0.323	10.077	1	0.002	2.784	1.479	5.238
	Common tumor site			25.693	4	0.000			
	Common tumor site (1)	1.945	0.635	9.382	1	0.002	6.994	2.015	24.279
	Common tumor site (2)	1.850	0.499	13.768	1	0.000	6.360	2.394	16.898
	Common tumor site (3)	0.508	0.504	1.015	1	0.314	1.662	0.619	4.468
	Common tumor site (4)	3.200	1.150	7.742	1	0.005	24.525	2.575	233.595
	T stage truncated			30.984	3	0.000			
	T stage truncated (1)	2.772	0.566	23.994	1	0.000	15.992	5.275	48.487
	T stage truncated (2)	2.369	0.487	23.628	1	0.000	10.687	4.112	27.779
	T stage truncated (3)	0.783	0.447	3.069	1	0.080	2.188	0.911	5.252
	Treatment modalities			19.057	4	0.001			
	Treatment modalities (1)	-1.927	0.507	14.474	1	0.000	0.146	0.054	0.393
	Treatment modalities (2)	-0.675	0.472	2.048	1	0.152	0.509	0.202	1.283
	Treatment modalities (3)	-1.934	0.597	10.496	1	0.001	0.145	0.045	0.466
	Treatment modalities (4)	-0.588	0.706	0.694	1	0.405	0.555	0.139	2.215
	Constant	-0.163	0.694	0.055	1	0.814	0.849		

[a] Variables(s) entered on step 1: Gender, Age groups at first diagnosis, ASA, Smoker, Common tumor sites, T stage truncated, UICC truncated, p16_Kat2, Treatment modalities.

Table S4. Results of binary logistic regression including response status at last follow up for the functional domain **pain**. Odds ratios (Exp(B)) with 95% confidence intervals are provided. Reference groups were: Gender female, age group >80 years; ASA III/IV; smoking <10 PY; alcohol consumption <daily; tumor stage T4, tumor site hypopharynx; treatment modality primary ST/RT; p16 negative.

		**B**	**S.E.**	**Wald**	**df**	**Sig.**	**Exp(B)**	**95%CI for Exp(B)**	
								**Lower**	**Upper**
**Step 1**[a]	ASA (1)	1.062	0.323	10.797	1	0.001	2.892	1.535	5.450
	T stage truncated			8.480	3	0.037			
	T stage truncated (1)	0.029	0.723	0.002	1	0.968	1.029	0.249	4.248
	T stage truncated (2)	1.113	0.588	0.037	1	0.847	1.120	0.354	3.543
	T stage truncated (3)	-1.082	0.547	3.907	1	0.048	0.339	0.116	0.991
	N stage truncated			0.810	3	0.847			
	N stage truncated (1)	0.166	0.661	0.063	1	0.802	1.180	0.323	4.315
	N stage truncated (2)	0.431	0.735	0.344	1	0.558	1.538	0.364	6.493
	N stage truncated (3)	0.585	0.852	0.472	1	0.492	1.796	0.338	9.538
	UICC truncated			3.587	3	0.310			
	UICC truncated (1)	0.126	1.053	0.014	1	0.905	1.134	0.144	8.929
	UICC truncated (2)	-0.708	0.825	0.735	1	0.391	0.493	0.098	2.485
	UICC truncated (3)	0.653	0.755	0.748	1	0.387	1.921	0.437	8.440
	Common tumor site			4.059	4	0.398			
	Common tumor site (1)	1.341	0.777	2.978	1	0.084	3.824	0.834	17.538
	Common tumor site (2)	0.510	0.518	0.969	1	0.325	1.665	0.603	4.595
	Common tumor site (3)	0.389	0.577	0.456	1	0.500	1.476	0476	4.574
	Common tumor site (4)	-0.013	0.649	0.000	1	0.984	0.987	0.277	3.519
	Treatment modalities			4.407	4	0.354			
	Treatment modalities (1)	1.080	0.544	3.947	1	0.047	2.945	1.015	8.551
	Treatment modalities (2)	0.413	0.411	1.011	1	0.315	1.512	0.675	3.384
	Treatment modalities (3)	0.617	0.610	1.024	1	0.312	1.853	0.561	6.121
	Treatment modalities (4)	0.390	0.680	0.329	1	0.566	1.477	0.389	5.607
	Constant	0.598	0.612	0.955	1	0.328	1.819		
**Step 5**[a]	ASA (1)	1.059	0.300	12.425	1	0.000	2.884	1.601	5.194
	T stage truncated			11.314	3	0.010			
	T stage truncated (1)	0.626	0.496	1.594	1	0.207	1.871	0.707	4.948
	T stage truncated (2)	0.235	0.424	0.307	1	0.579	1.265	0.551	2.907
	T stage truncated (3)	-0.736	0.421	3.063	1	0.080	0.479	0.210	1.092
	Constant	1.389	0.348	15.965	1	0.000	4.012		

[a] Variables(s) entered on step 1: ASA, T stage truncated, N stage truncated, UICC truncated, Common tumor sites, Treatment modalities.

Table S5. Results of binary logistic regression including response status at last follow up for the functional domain **mood**. Odds ratios (Exp(B)) with 95% confidence intervals are provided. Reference groups were: Gender female, age group >80 years; ASA III/IV; smoking <10 PY; alcohol consumption <daily; tumor stage T4, tumor site hypopharynx; treatment modality primary ST/RT; p16 negative.

		B	S.E.	Wald	df	Sig.	Exp(B)	95%CI for Exp(B)	
								Lower	Upper
**Step 1**[a]	Gender (1)	0.834	0.351	5.659	1	0.017	2.302	1.158	4.576
	ASA (1)	0.531	0.326	2.645	1	0.104	1.700	0.897	3.222
	Constant	1.370	0.354	14.971	1	0.000	3.934		
**Step 2**[a]	Gender (1)	0.807	0.349	5.356	1	0.021	2.240	1.131	4.436
	Constant	1.718	0.290	35.019	1	0.000	5.571		

[a] Variables(s) entered on step 1: Gender, ASA.

Table S6. Results of binary logistic regression including response status at last follow up for the functional domain **neck and shoulder mobility**. Odds ratios (Exp(B)) with 95% confidence intervals are provided. Reference groups were: Gender female, age group >80 years; ASA III/IV; smoking <10 PY; alcohol consumption <daily; tumor stage T4, tumor site hypopharynx; treatment modality primary ST/RT; p16 negative.

		B	S.E.	Wald	df	Sig.	Exp(B)	95%CI for Exp(B)	
								Lower	Upper
**Step 1[a]**	ASA (1)	0.321	0.319	1.011	1	0.315	1.378	0.738	2.575
	Common tumor site			1.959	4	0.743			
	Common tumor site (1)	-0.784	0.758	1.070	1	0.301	0.456	0.103	2.017
	Common tumor site (2)	-0.508	0.699	0.529	1	0.467	0.601	0.153	2.368
	Common tumor site (3)	-0.222	0.822	0.073	1	0.787	0.801	0.160	4.013
	Common tumor site (4)	-0.219	0.748	0.085	1	0.770	0.804	0.186	3.480
	T stage truncated			0.449	3	0.930			
	T stage truncated (1)	0.438	0.665	0.434	1	0.510	1.550	0.421	5.703
	T stage truncated (2)	0.175	0.538	0.106	1	0.745	1.191	0.415	3.417
	T stage truncated (3)	0.214	0.525	0.166	1	0.683	1.239	0.443	3.467
	N stage truncated			2.201	3	0.532			
	N stage truncated (1)	-1.099	1.261	0.759	1	0.384	0.333	0.028	3.947
	N stage truncated (2)	-0.905	1.254	0.520	1	0.471	0.405	0.035	4.729
	N stage truncated (3)	-1.388	1.077	1.661	1	0.197	0.250	0.030	2.060
	UICC truncated			0.580	3	0.901			
	UICC truncated (1)	-0.054	1.004	0.003	1	0.957	0.947	0.132	6.778
	UICC truncated (2)	-0.530	0.819	0.419	1	0.517	0.588	0.118	2.931
	UICC truncated (3)	-0.174	0.707	0.060	1	0.806	0.841	0.210	3.359
	p16_Kat2 (1)	0.893	0.394	5.139	1	0.023	2.443	1.129	5.287
	Treatment modalities			6.922	4	0.140			
	Treatment modalities (1)	1.344	0.675	3.971	1	0.046	3.836	1.022	14.396
	Treatment modalities (2)	0.637	0.656	0.942	1	0.332	1.890	0.523	6.837
	Treatment modalities (3)	0.161	0.757	0.045	1	0.831	1.175	0.266	5.183
	Treatment modalities (4)	0.127	0.624	0.041	1	0.839	1.135	0.334	3.855
	Constant	2.336	1.395	2.803	1	0.094	10.338		
**Step 6[a]**	p16_Kat2 (1)	0.833	0.354	5.543	1	0.019	2.301	1.150	4.606
	Treatment modalities			10.768	4	0.029			
	Treatment modalities (1)	1.433	0.615	5.423	1	0.020	4.192	1.255	14.005
	Treatment modalities (2)	0.724	0.604	1.437	1	0.231	2.063	0.631	6.742
	Treatment modalities (3)	0.217	0.693	0.098	1	0.754	1.242	0.319	4.830
	Treatment modalities (4)	0.240	0.563	0.181	1	0.671	1.271	0.421	3.834
	Constant	0.962	0.522	3.396	1	0.065	2.618		

[a] Variables(s) entered on step 1: ASA, Common tumor sites, T stage truncated, N stage truncated, UICC truncated, p16_Kat2, Treatment modalities.
